# Head computed tomographic measurement as a predictor of outcome in patients with subdural hematoma with cerebral edema

**DOI:** 10.1186/s13049-016-0271-y

**Published:** 2016-06-07

**Authors:** Hitoshi Yamamura, Takasei Morioka, Tomonori Yamamoto, Yasumitsu Mizobata

**Affiliations:** Department of Critical Care Medicine, Graduate School of Medicine, Hirosaki University, 5 Zaifuchou, Hirosaki city, Aomori 036-8562 Japan; Department of Critical Care Medicine, Graduate School of Medicine, Osaka City University, 1-4-3 Asahimachi, Osaka City, 545-8585 Japan

## Abstract

**Background:**

The ability to predict outcome in patients with cerebral edema is important because it can influence treatment strategy. We evaluated whether differences in head computed tomographic (CT) measurements in Hounsfield units (HU) of white matter and gray matter can be used as a predictor of outcome in patients with subdural hematoma with cerebral edema.

**Methods:**

We evaluated 34 patients who had subdural hematoma with cerebral edema following acute closed head trauma and had undergone head CT within a few hours of admission. We divided them into the survival (n = 24) group and death (n = 10) group, and measured the HU of white matter and gray matter at injury and non-injury sites.

**Results:**

There were no significant differences in operation time or blood loss during surgery between the two groups. Only the HU of white matter in the injury site of patients in the death group were decreased significantly. A cut-off value of 31.5 for HU of white matter showed 80.0 % sensitivity and 99.9 % specificity for death; the area under the curve was 0.91.

**Discussion:**

Our results are more evidence of the support of neurogenic edema in trauma rather than an important clinical tool at this stage. However, HU values in WM may be one factor in the decision-making process that affects patient outcome. Changing the treatment strategy in patients with a low HU value in the WM at the injury site may bring about an improvement in patient outcome.

**Conclusion:**

Measurement in HU of white matter at the injury site might be useful as a predictor of outcome in patients with subdural hematoma with cerebral edema.

## Background

Severe brain injury results in high mortality and morbidity in trauma patients. If excessive brain edema occurs, it can become uncontrollable and cause secondary brain damage. We experienced differences in the severity of the brain edema even if the thickness of the hematoma was the same, and this difference was associated with outcome. The assessment of traumatic cerebral edema is difficult. Vasogenic edema caused by the destruction of the blood brain barrier and increased vascular permeability are a result of serum ingredients leaking outside the capillaries. The most frequent cause of traumatic brain edema is vasogenic, and direct brain cell damage induces cytotoxic edema. The edema induced by severe head injury such as subdural hematoma is mixed edema, not only vasogenic but also cytotoxic. Previous studies have reported on the assessment of brain edema using quantitative computed tomography (CT) [1–6], perfusion CT [7–9], magnetic resonance imaging in animals [10,11] and in humans [12], and transcranial Doppler [13]. However, there are few reports that correlate cerebral edema with the prediction of outcome. Von Holst et al. [14] reported that the Hounsfield units (HU) of white matter (WM) in patients with head trauma correlated with specific gravity values measured in surgical specimens. The ability to predict outcome in patients with cerebral edema is important because it can influence treatment strategy.

This study aimed to evaluate whether differences in head CT measurements in HU of WM and gray matter (GM) can be used as a predictor of outcome in patients with subdural hematoma with cerebral edema.

## Methods

### Study population

We evaluated 34 patients who had subdural hematoma with cerebral edema and had undergone head CT within a few hours of admission to the critical care unit of Osaka City University Hospital for acute closed head trauma from January 2008 to December 2013. Exclusion criteria were WM diseases such as cerebral infarction, dementia, depression, or other established damage. We divided the patients into two groups according to outcome: the survival group (*n* = 24) and the death group (*n* = 10). This study was a retrospective review of patient records and imaging files obtained from the hospital patient database.

### CT assessment

CT images were obtained on a SOMATOM CT system (Siemens, Germany). All CT images were non-contrast-enhanced 5-mm axial sections obtained parallel to the orbitomeatal baseline at 135 kV and 150 mA. Images were viewed on a 512 × 512-pixel monitor on an electrical communication system. We obtained standardized HU measurements of the WM and GM at 6 points: the injury and non-injury site at each of the frontal, temporal, and occipital lobes, while avoiding the site of brain hemorrhage. Two radiologists standardized the placement of the measurements at the basal ganglia level.

**Fig. 1:**
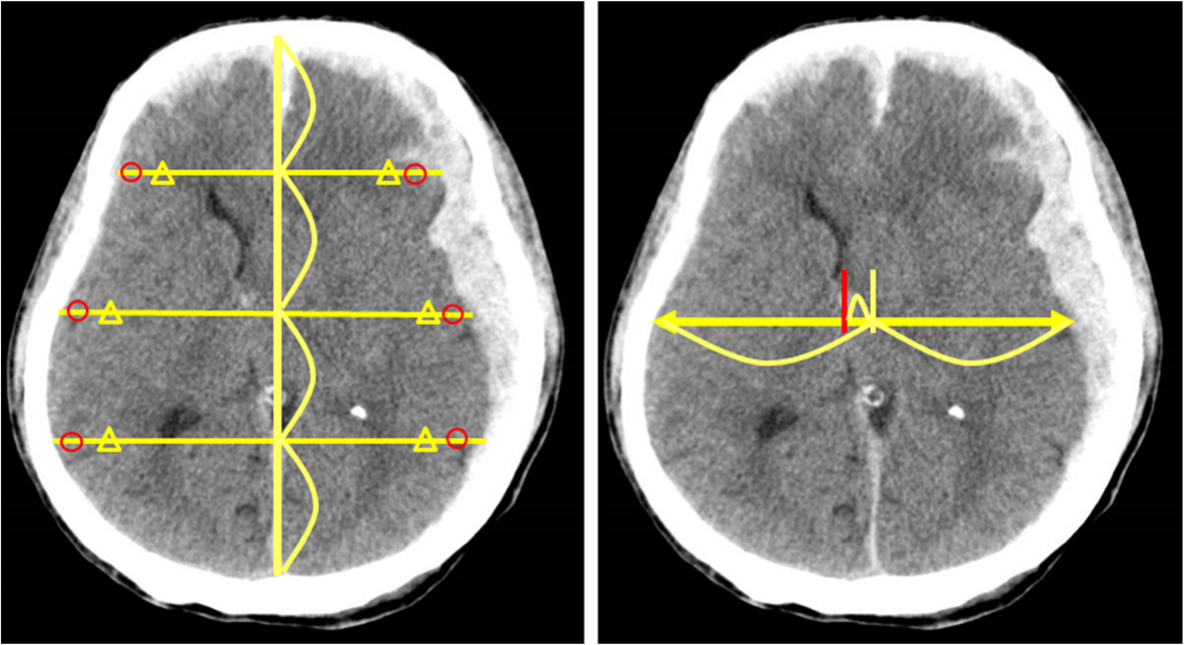
The measurement for Hounsfield units of grey matter (circle) and white matter (triangle) (left panel). The measurement for the displaced distance from the median (right panel)

The radiologist drew a centerline from the frontal pole to the occipital pole and placed hallmarks at the quarter, halfway, and three-quarter points to divide the centerline length into 4 equal parts. The radiologist then drew lines perpendicular to the centerline to the right and left of these three hallmarks and placed regions of interest (ROIs) (10 mm [2]) along these lines at 10 mm inside the cranial bone or hematoma on either side as three measurement points of the GM and at 15 mm inside of these points as three measurement points of the WM (Fig. 1). The HU values of the WM or GM were calculated as the mean HU value of the WM and mean HU value of the GM. We also measured the displaced distance from the median (DDM), which is the difference between the centerline of the line and the centerline of the anatomical mark (Fig. 1). We assessed the correlation between outcome and HU measurements of the WM and GM or DDM.

### Statistical analysis

Results are expressed as the mean ± standard deviation (SD) or the median (range). Differences in continuous variables between the two groups were compared using the Student unpaired *t*-test when the variable showed a normal distribution or the Mann–Whitney U-test when it did not. Using the receiver operating characteristic curve (ROC) method, we calculated the sensitivity, specificity, and area under the curve (AUC) to determine the diagnostic accuracy of our findings. The kappa value was used to analyze the data to examine the reliability of the radiologist to make the assessments. A kappa value of 0–0.20 was considered as slight agreement; 0.21–0.40 as fair; 0.41–0.60 as moderate; 0.61–0.80 as substantial; and 0.81–1 as almost perfect agreement [15]. A probability value of *p* < 0.05 was considered statistically significant. Statistical analyses were performed using JMP Version 10.0.2 for Windows (SAS Institute, Cary, NC).

## Results

Patient characteristics are presented in Table 1. There were significant differences between the survival and death groups with respect to the Glasgow Coma Scale (GCS), systolic blood pressure (SBP), and initial levels of fibrin/fibrinogen degradation products (FDP). In the survival group, 4 of the 12 patients experiencing falls suffered self-inflicted falls. In the death group, 1 patient’s injury was associated with pelvic fracture, whereas all of the other patients suffered head injury alone. In the survival group, 9 patients had an associated injury such as rib fracture, 5 had lung contusions, 2 had cervical spine injury, and the others suffered injuries to the face or extremities.

**Table 1:** Patients Demographics and Characteristics

Characteristic	Survival group (*N* = 24)	Death group (*N* = 10)	*P* value
Age, mean (SD), years	59 (15)	66 (35)	0.23
Sex			
Female	7	6	
Male	17	4	
Mechanism of injury			
Motor accident	9	7	
Fall	8	2	
Others	7	1	
AIS scoring, mean (SD)	32 (16)	26 (8)	0.91
Initial SBP, mean (SD), mmHg	152 (38)	183 (11)	0.02
Initial HR, mean (SD), min-1	86 (21)	88 (23)	1.00
Initial GCS, mean (SD)	10 (3)	6 (4)	0.01
Initial FDP, mean (SD), μg/mL	83 (64)	305 (252)	0.01
Initial lactate, mean (SD), mmol/L	3 (1.1)	2 (0.4)	0.39
Initial base excess, mean (SD)	0 (1.8)	0 (1.1)	0.80
Time from injury to CT examination, mean (SD), min	68 (21)	52 (17)	0.12
Time from injury to operation, mean (SD), min	144 (77)	168 (121)	0.78
Operation			
External decompression	17	10	
Hematoma removal	18	10	
Neurological prognosis			
GOS (2)	5		
GOS (3–5)	19		

*AIS* Abbreviated Injury Score, *SBP* systolic blood pressure, *HR* heart rate, *GCS* Glasgow Coma Scale, *FDP* fibrin/fibrinogen degradation products, *CT* computed tomography, *GOS* Glasgow Outcome Scale

Values are mean (SD) or number

The time from injury insult to the performance of surgery was not significantly different between the survival group and death group. External decompression and hematoma removal were carried out in all 10 patients in the death group and in 17 patients in the survival group. Hematoma removal only was performed in one patient in the survival group. The cause of death in all patients in the death group was brain herniation due to brain edema. Average time from admission to death was 4.7 days. We did not perform post mortem examinations or autopsy imaging.

In the survival group, 6 patients did not undergo surgery, and the outcome in all 6 was good recovery (Glasgow Outcome Scale: 5). Surgery was performed in 28 of the patients in the survival group. There were no significant differences in operation time (*p* = 0.38) or blood loss (*p* = 0.35) during surgery between the survival group and death group. However, infusion volume during surgery in the death group was higher than that in the survival group.

**Fig. 2:**
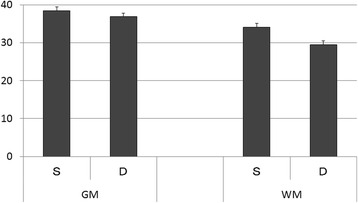
Hounsfield units (HU) of the injury site. The value of HU of white matter in the survival group was significantly higher than that in the death group (33.9 [2.7] vs. 29.5 [3.4] HU) (*p* <0.01)

**Fig. 3:**
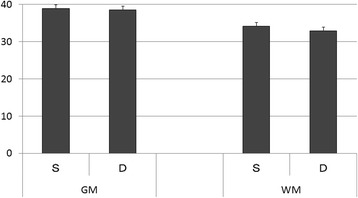
Hounsfield units of non-injury sites. There was no significant difference in the value of Hounsfield units of the grey matter or white matter between the survival group and the death group

**Fig. 4:**
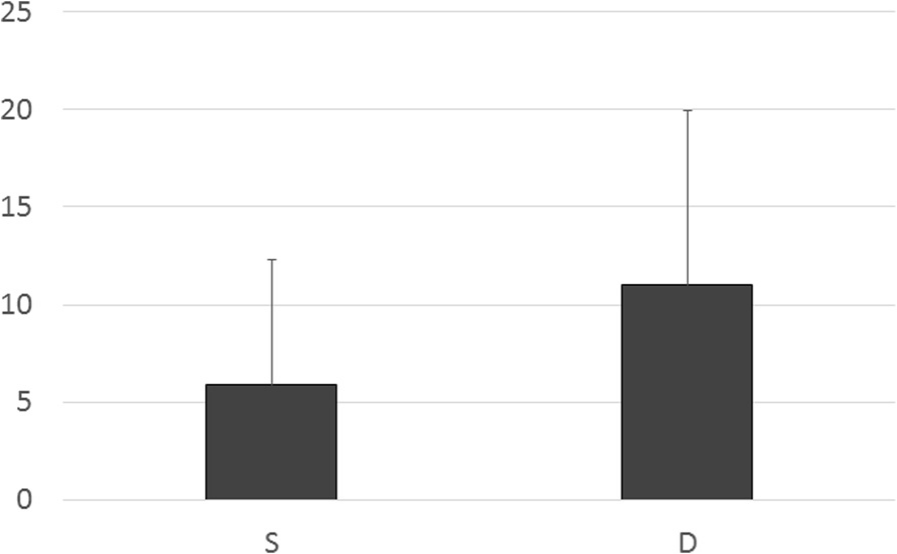
Results of the displaced distance from the median. There was no significant difference in the displaced distance from the median between the survival group and the death group

**Fig. 5:**
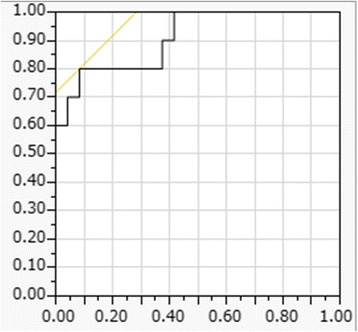
Receiver operating characteristic curve analysis for Hounsfield units of white matter at the injury site. A cut-off value of 31.5 for Hounsfield units of white matter showed 80.0 % sensitivity and 99.9 % specificity for predicting death. The area under the curve was 0.91

Inter-rater reliability of the two radiologists is presented in Table 2. The kappa value (k = 0.80–0.87) indicates substantial to almost perfect inter-rater reliability. The HU values of the injury or non-injury site are indicated in Figs. 2 and 3. Only the HU value of WM in the injury site of the death group was decreased significantly. There was no significant difference in the DDM between the survival group and death group (*p = 0.25*) (Fig. 4). We also reanalyzed the DDM-derived hematoma thickness. The hematoma thickness was 5.2 ± 1.2 mm in the death group and 3.8 ± 2.0 mm in the survival group. There was no significant difference in the DDM subtracted hematoma thickness between the survival group and death group (*p* = 0.45). However, the DDM in the death group was slightly higher than that in the survival group. The DDM did not correlate with the initial FDP, SBP, or GCS. ROC analysis for HU of WM of the injury site is shown in Fig. 5. A cut-off value of 31.5 for HU of WM showed 80.0 % sensitivity and 99.9 % specificity for death; the AUC was 0.91. Of the 24 surviving patients, however, only two had a HU value of less than 31.5.

**Table 2:** Inter-rater Reliability of the Two Radiologists

	*Radiologist 1*	*Radiologist 2*	*Kappa value (CI)*
Injured site GM, mean (SD)	38.1 (3.7)	37.8 (4.0)	0.85 (0.75–0.95)
Injured site WM, mean (SD)	32.6 (3.1)	33.0 (4.1)	0.87 (0.84–0.92)
Non-injured site GM, mean (SD)	38.4 (3.0)	39.1 (3.8)	0.79 (0.69–0.90)
Non-injured site WM, mean (SD)	33.5 (3.5)	34.1 (3.6)	0.82 (0.70–0.94)

*GM* gray matter, *WM* white matter, *CI* 95 % confidence interval

## Discussion

HU values measured at the initial CT were found to be the best predictor of outcome in patients with subdural hematoma due to brain edema. To the best of our knowledge, this is the first study to assess brain edema due to head trauma with subdural hematoma using head CT measurement expressed in HU of GM or WM. Especially, a cut-off value of 31.5 HU showed sufficient accuracy in discriminating between patients who survived and those who died. The HU values in the whole WM of patients with severe micro-angiopathic disease, demyelination or leukodystrophy/encephalopathy are decreased. In the immediate period after an injury such as acute subdural hematoma, there is a difference in the HU level of the WM between the injured side and non-injured side. In this study, the HU level of the non-injured side was 37.6 in the death group and 39.2 in the survival group. This finding is different between an injury and the above diseases.

We believe that the damage to brain tissue was much higher in the death group than in the survival group because both the initial FDP and SBP of the patients in the death group were higher than those of the patients in the survival group. These results may indicate that intracranial pressure in the death group was higher than that in the survival group at admission. However, we did not measure intracranial pressure in the acute phase in these patients.

Interestingly, traumatic brain injury caused an increase in the water content of the WM. WM edema has been observed and previously reported in relation to several diseases [16]. In post-cardiac arrest patients with brain edema, the HU values of WM showed no relative change in the hypoxic group, but the HU values decreased in the GM [17–20]. Changes in HU values depend on the water content of the brain tissue. There is more water content in the GM than in the WM following a hypoxic insult to the brain that results in edema, and such edema is more often present in the cerebral cortex than in the WM [16]. In brain cell death, the fatty myelin does not change during the acute phase; however, the intra- and extracellular water content does change after cell death. The results of the present study are controversial in terms of the changes caused by traumatic brain injury. Our finding that HU values on head CT scans decreased in the WM at the injury site within a very short time following traumatic insult was interesting. The midline shift was greater than the thickness of the hematoma, meaning that the brain swelling was related to brain damage. Bartels reported that in patients with a traumatic acute subdural hematoma a clear correlation between midline shift and thickness of the hematoma was found [21]. All patients with midline shift exceeding the thickness of the hematoma by 3 mm or more died. This report supported our results of the brain swelling was related to brain damage.

Klatzo divided brain edema into two categories: vasogenic edema and cytotoxic edema [22]. In clinical situations, these two types of edema are mixed, and a distinction between them cannot be made. The mechanism of cytotoxic edema is characterized by the expansion of the brain cells without the occurrence of vascular breakdown. In contrast, the mechanism of vasogenic edema is characterized by the destruction of capillary vessels; morphologically, this occurs due to the expansion of both external cell cavities in the WM and astrocyte cells [23]. Generally, cytotoxic brain edema is caused by hypoxia, metabolic disorders, water intoxication, or the early phase of brain infarction. Vasogenic brain edema is caused by brain injury, encephalitis, and the late phase of brain infarction. Recently, Barzó et al. suggested that a new, third category of edema—neurotoxic—may contribute to increased water content in the tissue following trauma [10]. First, there is an increase in brain water content and the water diffusion distance during the first 40 to 60 min post injury, indicating an increase in the volume of extracellular fluid. Second, a more widespread and slower formation of edema caused predominately by cellular swelling begins within 1 h post injury and becomes dominant at 1 or 2 weeks post injury. In our study, CT examination was carried out about 1 h after brain injury, and brain edema was already present, which supports our results. Several reports have discussed the correlation between prediction and head CT findings in patients with traumatic brain injury. Bullock et al. measured the specific gravity of WM using a micro-gravimetric method in 20 comatose patients [24]. Their results showed a significant correlation between the specific gravity values and CT density numbers and that cerebral vascular engorgement is the cause of the high specific gravity. Von Holst et al. measured the water content of the brain in specimens of WM obtained surgically in patients with mixed head injury patterns of focal and diffuse injury, and an HU value of 30.5 was found on CT images in the WM of the brain injury patients [14]. The distinction of GM and WM on CT is made at distances that will not always represent the GM/WM junction. Herniation, cerebral edema, and established damage can affect this. Other experiments examined the water content of traumatic brain edema using MR imaging in rats [10]. The water content of the brain continued to increase during the first 24 h post injury and at 45 min post injury, the water diffusion distance begins to decrease because of the slower and more widespread formation of cellular edema.

Although there are notable studies analyzing quantitative brain CT images in cerebral edema or traumatic brain injury, most of the prior studies on the quantitative assessment of cerebral edema used mean HU values in a pre-designated edematous region as the subject of analysis. Yuh et al. demonstrated that several predictors of outcome, including midline shift, cistern effacement, subdural hematoma volume, and GCS score are interrelated [3]. Kim et al. also measured HU values using unenhanced CT in pediatric patients with head trauma and assessed the correlation between HU value and the severity of cerebral edema [1]. They found that the HU value correlated highly with the existence of severe cerebral edema, and the significant difference between deceased and surviving patients was related to the proportion of pixels with HU values of 19 to 23. In another head CT study of cerebral edema, Huang et al. reported that contrast enhancement in head CT was highly associated with the progression of hemorrhage and greater volume of edema at 24 h [5].

In these previous studies using quantitative CT, the objective was to measure the HU values of the whole cerebrum, hematoma volume, or cistern effacement [3]. Further, these studies used special programs to determine prognostic value. In our study, however, few errors occurred because we chose measurement positions that avoided hematoma.

There are some important limitations in this study. First, this is a retrospective, single-institution study with small sample size. Therefore, to definitively confirm our results, a future prospective, multicenter study with a larger sample size would need to be conducted to obtain conclusive evidence of our findings. Second, evaluation with HU values is not reliable when bleeding is present in the measurement locations. However, we chose the placement of ROI’s to avoid hematoma or brain contusions. Third, physician judgment and operative time might have had an effect on convalescence. However, we used our standardized protocol for the treatment of head trauma including subdural hematoma, and as a result, we could plan a unified treatment policy. Fourth, the measurement to set the ROI was difficult. However, we examined the location of grey and white matter measurement the distance that maintained the same from a brain described in method. Fifth, intracranial pressure (ICP) could not be measured in all cases at the time of the first CT image. Thus, we could not evaluate the relation between ICP and HU, and we cannot explain a possible relation of the ICP with the distances measured on CT. The strategy for the treatment of subdural hematoma is affected by many factors, such as age, anticoagulation, persisting disease, and other associated injury. Our findings are more evidence of the support of neurogenic edema in trauma rather than an important clinical tool at this stage. However, HU values in WM may be one factor in the decision-making process that affects patient outcome. Changing the treatment strategy in patients with a low HU value in the WM at the injury site may bring about an improvement in patient outcome.

## Conclusions

The findings of this study suggest that the measurement of HU values in WM at the injury site might be useful as a predictor of outcome in patients with subdural hematoma with cerebral edema. An effective treatment plan needs to be formulated in high-risk patients in whom the HU value of WM is below 31.5.
